# Profile of differentially expressed Toll-like receptor signaling genes in the natural killer cells of patients with Sézary syndrome

**DOI:** 10.18632/oncotarget.21006

**Published:** 2017-09-18

**Authors:** Kelly C.G. ManfrereC, Marina P. Torrealba, Denis R. Miyashiro, Nátalli Z. Pereira, Fabio S.Y. Yoshikawa, Luana de M. Oliveira, Jade Cury-Martins, Alberto J.S. Duarte, José A. Sanches, Maria N. Sato

**Affiliations:** ^1^ Laboratory of Medical Investigation, LIM-56, Department of Dermatology, Tropical Medicine Institute of São Paulo, University of São Paulo Medical School, São Paulo, Brazil; ^2^ Cutaneous Lymphoma Clinic, Hospital das Clínicas, Department of Dermatology, University of São Paulo Medical School, São Paulo, Brazil

**Keywords:** Sézary syndrome, natural killer cells, Toll-like receptor 7/8, memory NK cells, differentially expressed genes

## Abstract

Sézary syndrome (SS), an aggressive and leukemic form of cutaneous T-cell lymphoma, usually results in shortened survival. Improving innate immunity in SS by targeting natural killer (NK) cells with Toll-like receptor (TLR) agonists could be an interesting modulatory strategy. We evaluated the NK cell populations in SS patients assessing activating and inhibitory receptors expression and profiled the differential expression of TLR signaling pathway genes in unstimulated NK cells and after TLR7/8 stimulation. We observed preserved CD56^bright^ NK cells and a low percentage of CD56^dim^ NK cells in the peripheral blood of SS patients compared to those in the healthy control group. Both NK cell populations showed down-modulation of NKG2C and NKG2D expression, which was associated with high serum levels of the soluble form of NKG2D ligands. In contrast, an expansion of “memory” CD57+ NKG2C+ NK cells and high cytomegalovirus antibody titers were detected in SS patients. Profiling of the TLR signaling genes in NK cells from SS patients showed an abundance of differentially expressed genes (DEGs) in NK cells in the unstimulated condition, with mostly up-regulation of NFκB/JNK p38 pathway genes, but there was down-regulation of type I (IFN-α/β) and II (IFN-γ) interferon and IL-12A. After activation of NK cells with TLR7/8 agonist, the down-regulated genes correlated with the IFN response, and IL-12 became up-regulated, together with other antitumor factors. NK cell activation with a dual agonist for TLR7 and TLR8 is able to induce the expression of IFN-γ and type I IFN, which can improve immunity in SS patients.

## INTRODUCTION

Sézary syndrome (SS) is an aggressive and advanced form of CTCL with circulating malignant T cells. Impaired immunity in SS [1, 2] probably is crucial for disease progression [3]. Investigation how to improve SS immunity may contribute to therapeutic strategies.

Natural killer (NK) cells are cytotoxic type 1 innate lymphoid cells with potential for cancer immunotherapy and for treating viral infections. There are two NK cell populations: CD56^dim^ CD16^bright^NK cells are predominant in the peripheral blood, whereas NK cells from secondary lymphoid tissue and from other tissues are primarily CD56^bright^ NK cells [4]. Moreover, *in vivo*, human CD56^bright^ NK cells may undergo progressive differentiation toward CD56^dim^ NK cells [5].

Decreased NK cell activity has been described in CTCL [6–8], whereas despite the quantitative and qualitative defects, the NK cells in SS could exert potentially cytotoxic effects against Sézary cells [7, 9].

Major NK cell-activating receptors involved in cancer cell recognition include NKG2D. This activating immune receptor has been identified on NK cells, CD8 T cells, NKT cells, and subsets of γδ T cells [10]. NKG2D expression is variable on NK cells, and in some SS patients, expression is intensely down-regulated [11]. Human MHC class I chain-related genes (MICA and MICB) and ULBP1-5 have been recognized as ligands of NKG2D. At advanced tumor stages, sustained surface expression of NKG2D ligands and shedding of sMICA induces internalization and degradation of NKG2D, thus promoting tumor immune evasion [12]. Some Sézary cells express MICA, MICB and ULBP1 [11], as well as the soluble form of MICA (sMICA), as a possible mechanism to escape the immune system.

Expansion of an NK cell subset carrying an activating receptor heterodimer, comprising CD94 and NKG2C (CD94/NKG2C) could be elicited by human cytomegalovirus (HCMV) infection [13, 14]. CD57+NKG2C+ NK cells seem to identify the final stages of peripheral NK cell maturation; the number of these cells increases with age, and they exhibit “memory-like” features [15–17]. To date, there is no evidence of the presence of NK cells expressing NKG2C and/or a memory profile in SS or in mycosis fungoides (MF), despite the fact that CMV seropositivity is highly associated with MF and SS [18].

The scientific and clinical interest in TLR7 and TLR8 in cancer biology has originated from the antitumor activities of these molecules in preclinical models [19]. Imiquimod is widely used as a topical treatment for cutaneous tumors, including basal cell carcinomas [20, 21], keratoacanthomas [22, 23], actinic keratoses [24–26], and cutaneous metastases of melanoma [27, 28]. Moreover, imiquimod has been successfully used for the treatment of patch- and plaque-stage MF [29–31]. Imiquimod preferentially activates TLR7, but it exerts weak agonistic activity by TLR8 [32]. Synthetic substances, such as resiquimod (R848, ligand for TLR7/8), have been extensively studied either as single agents in experimental cancer models or as vaccine adjuvants in clinical trials [33, 34]. Resiquimod induces more pronounced cytokine secretion, macrophage activation and cellular immune responses than does imiquimod [35, 36]. Topical resiquimod can promote disease regression and enhance T-cell effector functions in cutaneous T-cell lymphoma [37]. Approximately 50% of patients exhibited activation of circulating dendritic cells and NK cells, indicating a systemic immune response, and untreated lesions typically regressed.

An attempt to enhance NK cell function using synthetic agonists of TLRs has been explored in SS. The combined effects of synthetic oligodeoxynucleotides (ODN) with the CpG motif (TLR 9 agonist) and IL-15 on the activation of NK cells in patients with CTCL led to induced activation of NK cells and enhanced activation of CD8 T cells [38]. The CD158k/KIR3DL2 marker may be able to help identify and enumerate neoplastic T-cells in SS, even when present at low levels [39]. Moreover, interaction of KIR3DL2 with CpG ODN can directly promote malignant cell death in SS [40]. Previously we observed a dysfunctional cytokine response in the PBMCs of SS patients upon TLR agonists (TLR2-TLR9) stimulation, emphasizing the role of TLR7/8 agonists (CL097), which partially restored inflammatory cytokine and type I and type II interferon (IFN) secretion [41]. Evaluating the effector response and the differential gene profile in NK cells upon TLR7/8 stimulation on the adaptor and effector molecules and the downstream TLR pathway may lead to the development of novel immunotherapies to enhance immunity in SS patients.

Here, we propose to investigate innate immunity in SS by evaluating NK cell subsets, activation/inhibitory receptor expression, soluble NKG2D ligand levels, “memory” phenotype NK cells, and the effector response induced by a TLR7/8 agonist and differentially expressed genes (DEGs) related to the TLR signaling pathway.

## RESULTS

### Activation/inhibition receptor expression in natural killer cell subsets in SS

Disturbance of innate immunity has been described in SS, including a reduction of NK cells [8]. Despite the reduced number and function of NK cells, they are potential effectors cells against Sézary cells [7], which favors the development of strategies to enhance NK cells. Therefore, first we analyzed the expression of activating/inhibitory markers of NK cell subtypes from peripheral blood. Laboratory characteristics of SS patients are shown in Supplementary Table 1. The gating strategy for CD56+ NK cells is shown in Supplementary Figure 1.

**Figure 1:**
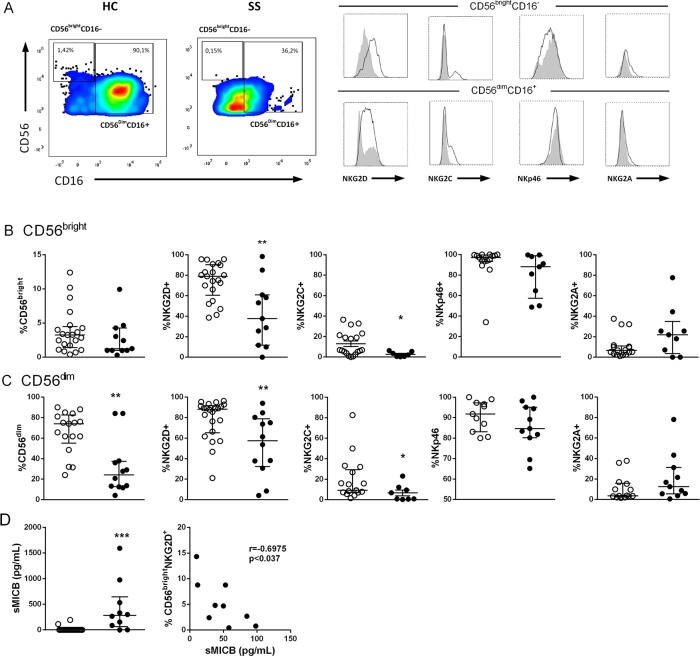
Impaired NKG2C/NKG2D expression in NK cell subsets of SS patients **(A)** Gate strategy for CD16+CD56^dim^ and CD16-CD56^bright^ NK cells for expression of activating/inhibitory receptor expression in peripheral blood by flow cytometry. Receptor expression of patient is represented with closed histogram and healthy donor with open histogram, **(B)** CD56^bright^ and **(C)** CD56^dim^ NK subsets from SS patients (n=9-12, closed circle) and healthy controls (HC, n=11-21, open circle) assessed for total number, NKG2D, NKG2C, NKp46 and NKG2A expression. **(D)** Soluble MICB (sMICB) levels were assessed in serum from SS (n=10, closed circle) and HC (n=22, open circle) subjects by means of ELISA, and correlation between CD16-CD56^bright^ NKG2D+ cells and sMICB levels of SS patients was assessed. The results are shown as medians and interquartile ranges (IQRs). ^*^p≤0.05, ^**^p≤0.01 compared with the HC group.

A reduced number of NK cells (Figure 1A) and CD56^dim^ NK cells (Figure 1C), was observed in the peripheral blood of SS patients while the number of CD56^bright^ NK cells number (Figure 1B) was not changed compared to HC group. Both NK cell subtypes CD56^bright^ (Figure 1B) and CD56^dim^ (Figure 1C) showed decreased NKG2D and NKG2C expressions in the SS group compared to HC group, with similar frequencies for NKp46 or NKG2A. We evaluated soluble MHC class I chain-related proteins A and B (sMICA/B) ligands of NKG2D in the serum of the patients. sMICA was detected in only 2/10 SS patients (SS7 and SS11), and sMICB was detected in 8/10 patients, at higher levels compared to HC samples (Figure 1D). A negative correlation was observed between the percentage of CD56^bright^NKG2D+ NK cells and sMICB levels, which may contribute to down-regulation of NKG2D expression in the NK cells of SS patients. Also, a decreased percentage of CD3+NKG2D+ cells was revealed by flow cytometry (Supplementary Figure 2).

**Figure 2:**
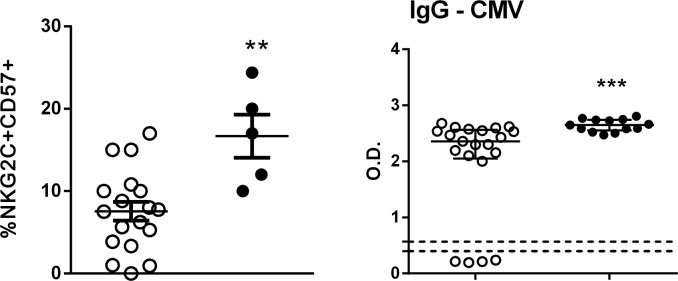
Determination of CD57+NKG2C+ NK cells Percentage of CD57+NKG2C+ in NK cells from peripheral blood of HC (n=18, open circle) and SS (n=5, closed circle) patients was assessed by flow cytometry. Serology for HCMV in serum from HC (n=21) and SS (n=13) groups was performed by ELISA. The results are shown as mean with SEM. ^**^p≤0.01, ^***^p≤0.001 compared with the HC group.

CD56+CD57+NKG2C+ NK cells have been termed “memory” NK cells, and these cells expand in response to infection with CMV [16]. Of note, only 5 SS patients analyzed showed an increased percentage of CD56+CD57+ NKG2C+ NK cells compared to HC subjects (Figure 2). All clinical evaluations were done with freshly obtained cells; at the time of the study, only 5 patients were available. Moreover, high CMV seropositivity was founded in SS patients (100%) as well as in HC (81%) subjects.

In addition, we analyzed the expression of NKG2C/NKG2D mRNA and their ligands in unstimulated PBMCs from both analyzed groups. Decreased NKG2C and NKG2D expression were verified in the PBMCs of SS patients (Supplementary Figure 3). Similar mRNA expression levels of ULBP-3 and MICA were observed in both groups, while a decrease in the expression of HLA-E, a ligand for NKG2C and NKG2A, was observed in the PBMCs from SS patients compared to those from HC subjects. The two SS patients that showed up-regulated MICA mRNA expression (SS7, SS11) are not the same patients that showed increased sMICA levels (SS4, SS8) in serum.

The data showed a decreased number of CD56^dim^ NK cells in the peripheral blood of SS patients, impaired expression of NK-activating receptors, such as NKG2D and NKG2C, and expansion of “memory” CD57+NKG2C+ NK cells, together with increased sMICA/MICB, which acts as tumor escape mechanism.

### NK cell response to a TLR7/8 agonist

Previously, we verified that TLR ligands have the ability to restore, at least partially, the expression of a broad range of cytokines and type I/III IFN in the PBMCs from SS patients [41]. We assessed the NK effector molecules, such as degranulating CD107a, IFN-γ and TNF, induced by TLR7/8 stimulation by means of flow cytometry; PMA plus ionomycin was used as a positive control.

**Figure 3:**
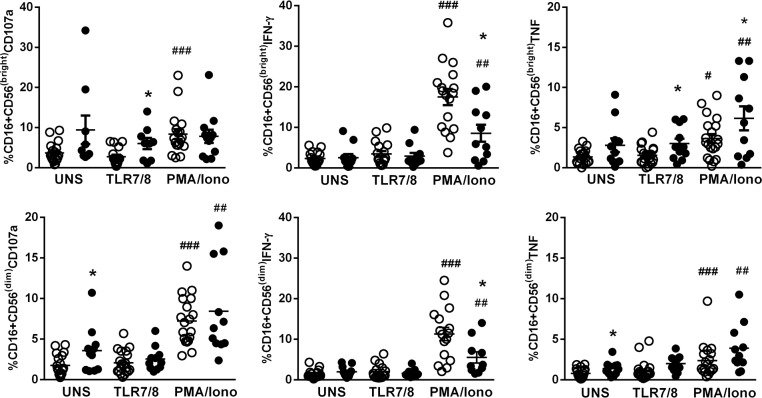
Effector molecules produced by NK cells after stimulation with a TLR7/8 agonist in SS patients CD56^bright^ and CD56^dim^ NK subsets from SS patients (n=11, closed circle) and healthy controls (HC, n=20, open circle) were assessed by flow cytometry for CD107a, IFN-γ, and TNF expression after stimulation with a TLR7/8 agonist (CL097) and PMA plus ionomycin. The results are shown as mean with SEM. ^*^p≤0.05, ^**^p≤0.01 compared with the HC group; ^#^p≤0.05, ^##^p≤0.01, ^###^p≤0.001 compared with the unstimulated condition.

Figure 3 shows an increased percentage of CD16+CD56^dim^NK cells in the unstimulated condition expressing CD107a and TNF in SS patients compared to HC subjects. After CL097 stimulation, CD56^bright^ NK cells demonstrated increased CD107a and TNF production compared to HC cells. A decreased IFN-γ response was detected in both NK cell populations with PMA plus ionomycin stimulation, while increased TNF secretion was observed in CD56^bright^ NK cells and decreased CD107a in CD56^dim^ NK cells in the SS group.

### Profile of the TLR signaling pathway in NK cells

A dysregulation in TLR signaling can compromise the immune response. Therefore, we evaluated the expression pattern of central genes in TLR-related transduction pathways upon activation with a TLR7/8 agonist (CL097). We assessed purified NK cells to verify the direct effect of TLRs activation on NK cells. By PCR array, 84 genes of the TLR signaling pathway (Supplementary Table 3) were assessed in purified NK cells from the peripheral blood of SS patients (SS9, SS2, and SS20) and HC subjects (n=three/per group) in the unstimulated condition and after stimulation with CL097. The three patients selected for our study were among the few who survived.

**Figure 4:**
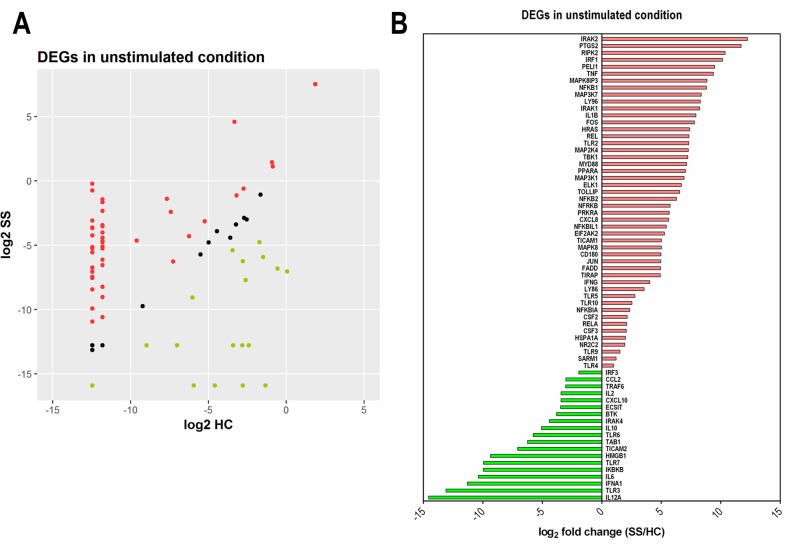
Abundance of differentially expressed genes (DEGs) of the TLR signaling pathway in NK cells from SS patients Freshly isolated NK cells were assessed in the unstimulated condition by PCR array for 84 genes using a human TLR signaling pathway kit. **(A)** Scatter plot of the normalized expression of DEGs (expressed as log2 of median values) in NK cells from SS patients compared to HC; genes were classified as increased (red), unchanged (black) or reduced expression (green). **(B)** Identity and fold change (SS in relation to HC, expressed as log2-fold change of median value) of individual DEGs. Data are representative of one experiment with three SS patients (SS19, SS2, and SS20) and three HC individuals (HC 1-3).

Figure 4 shows the profile of the differentially expressed genes (DEGs) in unstimulated NK cells. Among the 84 genes analyzed, 48 genes were over-expressed in SS patients in relation to HC, including several members of the NF-kB pathway and the JNK/p38 family (Figure 4A and 4B). There were 19 under-expressed genes, notably those related to the IFN pathway, such as IRF3, TLR7, IFNA1, TLR3 and IL-12A (Figure 4B). Moreover, only 17 genes showed similar expression between both groups. These findings show that even in the unstimulated condition, NK cells from SS patients have abundant differentially expressed TLR signaling genes.

**Figure 5:**
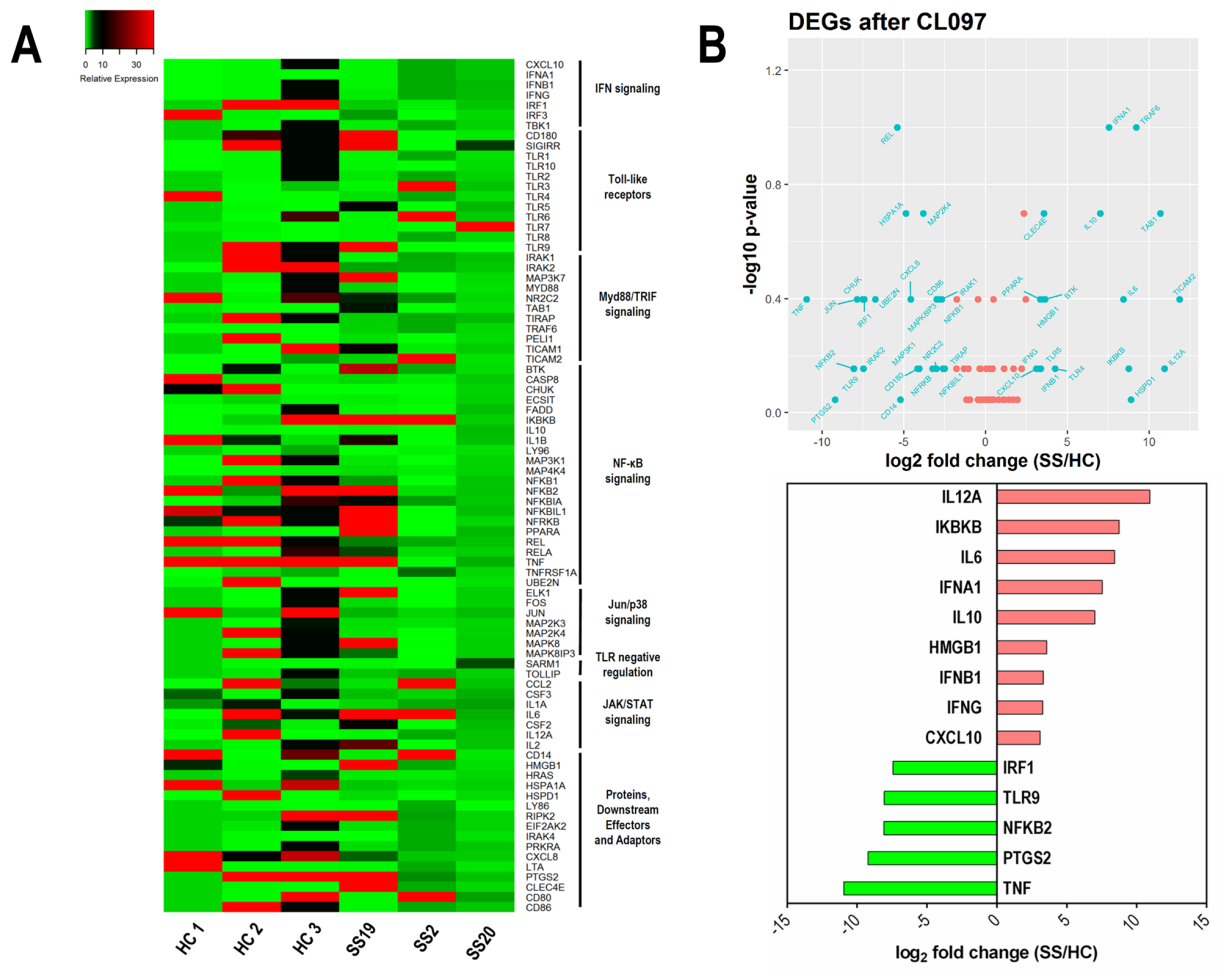
Up-regulation of IFN signaling-related genes in NK cells under TLR7/8 stimulation in SS patients **(A)** Heat map illustrating DEGs induced by stimulation with a TLR7/8 agonist on NK cells from SS patients (SS19, SS2, and SS20) and HC. Genes were clustered in signaling pathway subgroups. **(B)** Upper panel, Volcano plot of up- and down-regulated DEGs (log2-fold change against −log10 p-value); lower panel, ranking of top DEGs according to their fold change (SS compared to HC).

Figure 5A shows a heat map of the TLR signaling pathway genes after TLR7/8 stimulation in NK cells from SS and HC subjects. Again, a different profile of genes was detected in stimulated NK cells from SS patients, as shown by the volcano plot (Figure 5B), in which 28 genes were up-regulated in SS patients, 29 were down-regulated and 27 genes have similar expressions in both groups. Due to the clinical heterogeneity in SS patients, no statistical significance was detected in our analysis; however, the analyzed patients showed consistent expression profiles. Figure 5B shows some selected DEGs upon stimulation of TLR7/8. Notably, some genes that were repressed under normal circumstances in SS patients, especially those associated with IFN signaling, were up-regulated following CL097 stimulation, such as the cytokines IL-12A, IFNA1, IFNB1, IFN-γ and the IFN-γ-inducible chemokine CXCL10. In addition, the induction of IKBKB, which degrades the NFκB inhibitor IκB, may contribute to the activation of the NFκB pathway. In parallel, TLR7/8 stimulation also induced regulatory genes, such as IL-10, and anti-tumor factors, such as high-mobility group box 1 protein (HMGB1) [42].

The ratio between up/down-regulated genes changed from 2.52 in the unstimulated condition to 0.96 after TLR7/8 activation, revealing that in the unstimulated condition most of the DEGs were up-regulated in NK cells from SS patients.

## DISCUSSION

The innate lymphocytes NK cells are essential for innate immune responses against cancer and viral infections. Indeed, a genetic NK deficiency causes severe herpes virus infections and affects tumor clearance [43]. In SS, impaired immunity is one factor involved in disease progression. We verified that SS patients showed a reduced number of total NK cells and CD16+CD56^dim^ NK cells, while the CD56^bright^ NK population was preserved. The equilibrium percentage of CD56^bright^ NK cells in SS must be crucial in attempting to replace the number of terminally differentiated CD56^dim^ cells, which seems to be due to an impaired output of NK cells from bone marrow. It is known that the percentages and absolute numbers of NK cells increase during healthy aging, but the decrease in the CD56 bright subset is interpreted as a lower output of such NK cells from the bone marrow [44]. Moreover, the CD56^dim^ NK cell population is preferential for causing apoptosis in cancer [45].

NK cell receptors provide signals, which determine whether an NK cell becomes activated or inhibited. We detected a down-modulation of NK-activating receptors, such as NKG2D and NKG2C, regardless of NK population, while the inhibitory receptor NKG2A remained unchanged in SS subjects. In addition, decreased NKG2D mRNA expression was detected in the PBMCs from the SS group with wide NKG2D down-modulation, including by CD3+ T cells. Down-regulation of NKG2D could be due to factors such as TGF-β or metalloproteinases [46] and/or to NKG2D ligands, the production of which are a tumor evasion mechanism. TGF-β1 secreted by tumors is responsible for the poor NK lytic activity via down-regulation of NKG2D [47]. TGF-β serum levels in SS patients were similar to those in the HC group (data not shown), whereas increased serum sMICB levels were found in the majority of SS patients. The high sMICB serum levels found in SS patients could originate from several cells, including Sézary cells. In fact, MICB rather than MICA is highly expressed by neoplastic cells from CTCLs, including those in SS patients [48]. In addition, MICB transcript levels were strongly correlated to those of T-plastin [48], a factor characteristically expressed by neoplastic T-cells of CTCL [49]; the correlation suggests a relationship to blood tumor burden.

A broad variability of NKG2D expression has been described in some SS patients (2/6), some with severe down-regulation [11]. In our SS cohort, NKG2D expression was heterogeneous, and at least 50% of patients showed half the level of the control group. In addition, an inverse correlation between the percentage of CD56^bright^NKG2D+ NK cells and sMICB levels was detected. This finding suggests that NKG2D ligands may interact with receptors to induce internalization and degradation, contributing to its down modulation in SS patients.

An increased percentage of CD56+CD57+ NKG2C+NK cells was found in SS patients, together with high seropositivity to CMV. The subset of CD57+NKG2C+ NK cells has been described as exhibiting memory-like features and has potent effector functions [17]; HCMV infection is able to induce the expansion of CD94/NKG2C+ NK cells [13, 50]. CMV seropositivity is highly associated with SS and MF, even in the earliest stages of the disease, and is significantly higher than that of healthy and immune compromised controls [51]. The immunosuppressive status seems to be responsible for CMV reactivation [52]. Interaction of CD94/NKG2C with its ligand HLA-E was shown to drive the expansion of the CD94– NKG2C+ NK cell population [14]. We verified decreased HLA-E expression in the PBMC of SS patients, whereas we did not assess other cells from tissue, such skin cells. The expansion of this mature CD57+NKG2C+ NK subset detected in SS patients could be due to memory for CMV infection, and the fact that this population display potent function may be beneficial for the patients.

Employment of adjuvants such as TLR agonists has been widely studied in APCs and other cells, including NK cells. Human NK cells express TLR3, TLR7, and TLR8 [53]. Previously, we verified that the compound CL097, a TLR7/8 dual agonist, was able to induce type I and II IFNs and other cytokines in the PBMCs of SS patients [41]. For NK cells, we observed that activation through the TLR7/8 pathway induced mainly degranulating factor CD107a and TNF by CD56^bright^ NK cells. Therefore, to better investigate the NK response following TLR7/8 activation, we assessed DEGs related to the TLR signaling pathway in NK cells. We used purified NK cells to verify the direct role of TLR activation, and we did not split the NK cells into subtypes due to the scarcity of NK cells in patients with SS. Of note, unstimulated NK cells from SS patients showed abundant DEGs of the TLR signaling pathway (48/84 genes). These up-regulated genes included NFκB, JNK/p38 and MyD88-dependent genes and revealed the activated status of NK cells. Among the TLRs, the most intensively expressed was TLR2, which was possibly a consequence of bacterial infection; this is not surprising considering that sepsis is a common cause of death among SS patients. This aligns with a previous finding that some patients with SS showed up-regulation of TLR2 mRNA expression in unstimulated PBMCs [41].

Other up-regulated genes expression in unstimulated NK cells from SS patients were prostaglandin synthase 2 (PTGS2/COX2), which exerts a negative effect on NK cell function via PGE2 through PGE2 receptor EP4 [54]. Moreover, PGS2 is a growth factor for malignant T cells in MF [55]. The expression of some pro-inflammatory cytokines, such as TNF and IL-1β, were up-regulated in NK cells demonstrating again the activated profile of NK cells from SS patients. In contrast, it is possible that TNF can also exert a pathogenic role. Evidence based on MF skin lesions has shown that TNF is involved in MF tumorigenesis because a cDNA microarray study demonstrated the promotion of antiapoptotic signaling by the deregulation of TNF signaling and an autocrine TNF feedback loop [56].

Although DEGs were mostly up-regulated in NK cells from SS patients under normal conditions, the down-regulated DEGs included genes in the IFN pathway, such as IRF3, TLR7, IFNA1, TLR3 and IL-12A, and the IFN-γ-inducible chemokine, CXCL10. Importantly, NK cells seem to contribute to the deficit in IFN-γ, type I IFN and IL-12 genes, which are well characterized in SS patients [2, 29, 57], for whom IFN-α and IFN-γ are recommended as a treatment [58, 59].

Notably, the profile of DEGs after TLR7/8 stimulation showed that several inhibited genes became up-regulated, including those related to the type I and II IFN responses (IFNA1, IFNB1, IFNG, CXCL10) and IL-12 (IL-12A). We verified that CL097 was an interesting adjuvant to enhance the activity of NK cells in SS. Included in the NK genes up-regulated after CL097 activation was HMGB1, which can play an important role against tumor cells. NK cells invoke this strategy by employing HMGB1 protein, which specifically targets glycolysis in cancer cells and opens up new perspectives for cancer immunotherapy [42]. Moreover, NK cells, upon interaction with melanoma cells, can release a chemotactic form of HMGB1 protein that is capable of attracting additional activated NK cells [60]. Furthermore, another negatively regulated gene that becomes up-regulated in SS is IKBKB, which regulates the degradation of the NF-κB complex and favors the NF-κB-signaling pathway in NK cells.

However, some genes are down-regulated after stimulation, including IRF-1, a pivotal factor in the regulation of NK cells during infection, inflammation and metastasis [61], while PTGS2 is also down-regulated in NK cells, which could be favorable for NK function in SS.

Moreover, our data with TLR7-8 as adjuvant to potentiate the innate immunity, as NK cells, aligns with the data that topical resiquimod, an agonist of TLR7/8, is safe and effective in patients with stage IA-IIA CTCL, that can clear both treated and untreated skin lesions [37].

Our findings showed that NK cells in SS display different features than those in healthy subjects, with one aspect being decreased CD56^dim^NK population number, down-modulation of NKG2D, and impairment of IFN-γ response, and the other revealing a preserved CD56^bright^ NK population and the presence of mature, memory CD57+NKG2C+ NK cells. In addition, NK cells exhibited a constitutively activated status with abundant DEGs; after CL097 activation, up-regulation of crucial genes of the IFN response and anti-tumor response occurred. These findings emphasize the use of TLR agonists as adjuvants to improve innate immunity with NK cells in CTCL.

## MATERIALS AND METHODS

### Patients

We enrolled patients with Sézary syndrome (SS; n=13, 6 males, 7 females), with a median age of 61 years (range 48-84 years), from the Clinic of Cutaneous Lymphomas of the Department of Dermatology, Hospital das Clínicas, University of São Paulo Medical School, Brazil (HC/FMUSP). Diagnosis of SS was performed according to the revised clinical, histological, and biological criteria proposed by the International Society for Cutaneous Lymphomas (ISCL) and the cutaneous lymphoma task force of the European Organization of Research and Treatment of Cancer (EORTC) [62]. All SS patients have “de novo” erythrodermic; therefore, none of the cases were derived from MF progression. Healthy controls (HC; n=21, 10 males, 11 females), with a median age of 54 years (range 31-74 years), were selected from the Laboratory of Dermatology and Immunodeficiencies (LIM-56). Blood collection was performed before the initiation of treatment. Patients with dermatologic diseases, those using drugs, and those with a history of autoimmune diseases were not included in this evaluation. The exclusion criteria were treatment with immunosuppressant or immune-modifying drugs, pregnancy, and under 18 years of age. This study was approved by the São Paulo University Institutional Use Committee (CAAE, 07965312.0.0000.0068), and informed consent was obtained from all subjects. All experimental protocols within this study were performed in accordance with the Ethics Committee of this institution.

### Flow cytometry in peripheral blood

To analyze activation/inhibition markers in the NK cells, venous blood was collected in EDTA anticoagulant tubes, and staining was performed using the following antibodies: CD3 (BV605/clone SK7), CD19 (Horizon V500/clone HIB19), CD16 (APC-Cy7/clone 3G8), CD56 (Alexa 700/clone B159), NKG2D (PECy7/clone 1D11) NKp46 (APC/clone 9-E2) and CD57 (APC/clone NK-1) from BD Biosciences (San Jose, CA, USA) and NKG2A (PE/clone 131491) and NKG2C (Alexa Fluor 488/clone 134591) from R&D Minneapolis, MN, USA. Approximately 70 μL of whole blood was stained for 20 min and then incubated for 15 min with FACS lysing solution (BD FACS Lysing; BD Biosciences) to lyse the erythrocytes. After two washes in an isotonic solution (Hemoton SPEC; Brazil), 300,000 events were acquired using a flow cytometer (LSR Fortessa; BD Biosciences, USA) and were analyzed using FlowJo Software (Tree Star, Ashland, OR, USA).

### Flow cytometry for effector molecules in stimulated NK cells

Peripheral blood mononuclear cells (PBMCs) were isolated by centrifugation with Ficoll-Hypaque density gradient (GE Health Care, Uppsala, Sweden). PBMCs (1.0×106 cells/mL) were incubated with 5.0 μg/mL TLR7/8 agonist (CL097; InvivoGen, San Diego, CA, USA), phorbol myristate acetate (50 ng/mL) and ionomycin (1 μg/mL) and CD107a PE-Cy5 (Pe.Cy5/clone H4A3) to detect degranulating NK cells and incubated at 37°C with 5% CO2 for 6 h. After 2 h of incubation, Brefeldin (10.0 μg/mL; Sigma, St. Louis, MO, USA) was added to the cultures for another 4 h of incubation. After incubation, the cells were incubated with Live-Dead reagent (Invitrogen, Eugene, OR, USA) for 20 min at room temperature. Cells were then subjected to fixation with Cytofix/Cytoperm solution (BD Bioscience, San Diego, CA, USA) for 20 min and permeabilization with Perm/Wash solution for 20 min at 4°C. The cells were then stained with CD3 (BV605/clone SK7), CD19 (Horizon V500/clone HIB19), CD16 (APC-Cy7/clone 3G8), CD56 (Alexa 700/ clone B159), IFN-γ (Horizon V450/clone B27) and TNF (PE-Cy7/clone MAB11) antibodies. Next, the samples were washed with Perm/Wash buffer (BD Bioscience, San Diego, CA, USA) and diluted in isotonic solution. Fluorescence Minus One (FMO) controls were performed for all antibody panels to confirm proper compensation and define positive signals. A total of 300,000 events were collected and analyzed by flow cytometry (LSR Fortessa, BD Biosciences, USA).

### Real-time PCR

Total RNA was extracted from PBMCs using an RNeasyPlus Mini Kit (Qiagen, Valencia, CA, USA), and reverse transcription was performed with a Sensiscript Reverse Transcriptase Kit (Qiagen). The primers used in the real-time PCR assay for NKG2C, NKG2D, MICA, HLA-E, and UBLPB-3 are shown in Supplementary Table 2.

GAPDH mRNA levels were used to normalize the mRNA content from PBMCs. PCR was performed on an Applied Biosystems 7500 system using specific primers and SYBR Green (Applied Biosystems, Carlsbad, CA, USA) fluorescence detection reagents. The cycling protocol consisted of 10 min at 95°C, followed by 40 cycles of 15 s at 95°C and 60 s at 60°C. The amplification results were visualized and analyzed using Sequence Detection System (SDS) software (Applied Biosystems). Normalized expression was calculated as previously described by Livak [63].

### Determination of soluble NKG2D ligands by ELISA

Serum levels of ligands for the receptor NKG2D, such as MICA and MICB, were assessed by ELISA according the manufacturer recommendations (R&D System, Wiesbaden, German). Serology for HMCV was performed using a kit from Virion\Serion (Wurzburg, Germany).

### Toll-like receptor signaling pathway PCR array

Purified NK cells from the PBMCs of three SS patients (SS9, SS2, and SS20) and three HC subjects by cell sorting were left in RPMI medium supplemented with 10% human AB serum (Sigma Aldrich, EUA) for 18 h at 37°C. Cells were stimulated with a TLR7/8 agonist (5.0 μg/mL) or with medium only (unstimulated) for 4 hours at 37°C. The cells were stored in RNA later solution (Sigma Aldrich, EUA) at -80°C. Total cellular RNA was extracted from NK cells using an RNeasy Micro Kit (Qiagen). The first strand of cDNA was synthesized based on the instructions for the RT2 First Strand Kit and PreAMP kit (Qiagen, Valencia, CA, USA), and the final cDNA product was used for the RT2 Profiler PCR Array using SYBR Green-based real-time PCR according to the manufacturer's protocol (PAHS-018Z array, Qiagen). The array consisted ofa panel of 84 genes relevant to the TLR pathway (Supplementary Table 3) plus five housekeeping genes (B2M, HPRT1, RPL13A, GAPDH and ACTB), a genomic DNA control, three reverse transcription and three PCR quality controls. Only samples passing the PCR array run quality control, which assessed the absence of genomic DNA contamination and proper amplification of the reverse transcription controls and the positive PCR controls, were further evaluated. ΔCt was calculated as the difference between the Ct of the TLR pathway genes and the geometric average of the Ct of the housekeeping genes. Log-fold differences in expression were reported using the 2−ΔΔCt or 2−ΔCt method [63].

### Statistical analysis

The Mann-Whitney U-test was used to compare variables between the groups, the Wilcoxon matched pairs test was used to compare the baseline level with stimulated samples. p≤0.05 was considered significant.

## SUPPLEMENTARY MATERIALS FIGURES AND TABLES




