# Supplementary material for: Profile of differentially expressed Toll-like receptor signaling genes in the natural killer cells of patients with Sézary syndrome
Source: Oncotarget. 2017 Sep 18;8(54):92183–94. doi: 10.18632/oncotarget.21006 (PMC5696173; doi:10.18632/oncotarget.21006)
Supplement: Supplementary file 1 [file oncotarget-08-92183-s001.pdf]

Profile of differentially expressed Toll-like receptor signaling genes in the natural killer cells of patients with Sézary syndrome

SUPPLEMENTARY MATERIALS

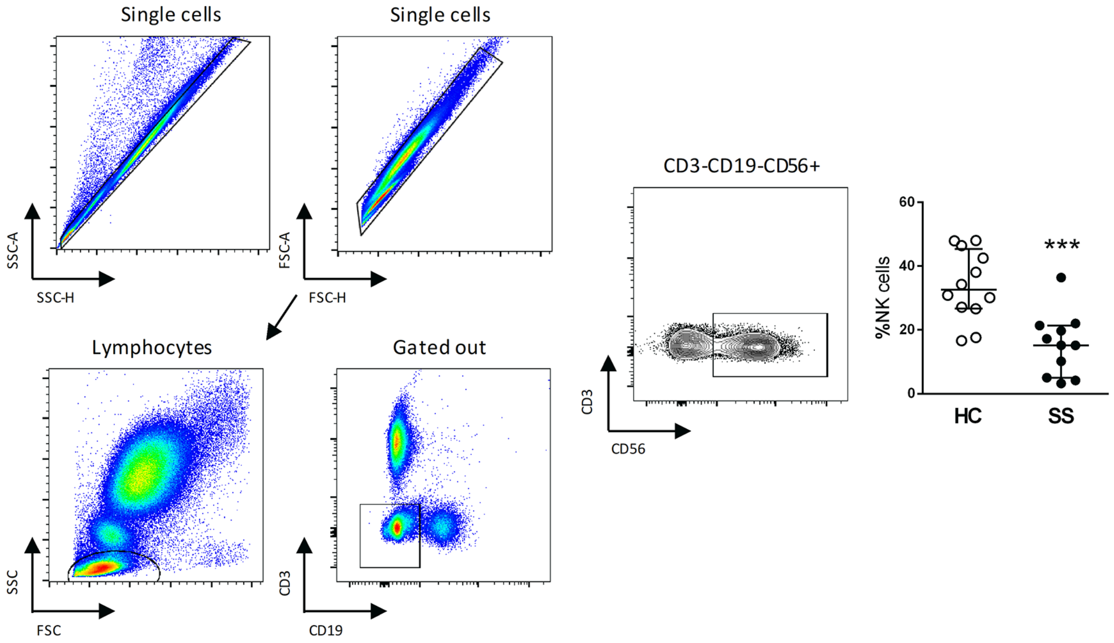

Supplementary Figure 1: Gating strategy for NK cells and total NK cell number. Gating strategy for NK cells. Percentage of CD3-CD19- CD56+ NK cells assessed by flow cytometry in HC (n=12, open circle) and SS (n=11, closed circle) patients. The result is shown as median and interquartile ranges (IQRs). ***p≤0.001 compared with the HC group.

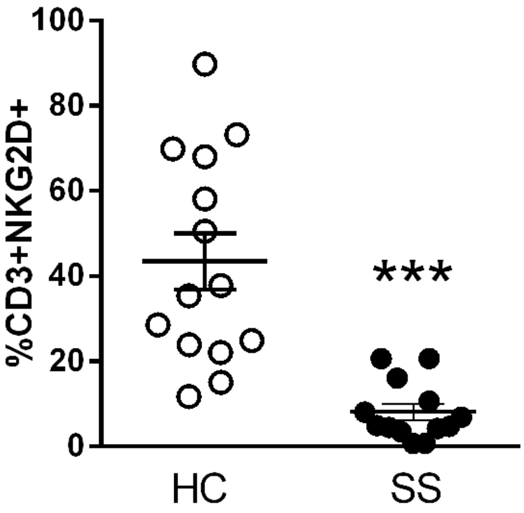

Supplementary Figure 2: Determination of CD3+ T cells expressing NKG2D. Percentage of CD3+ NKG2D+ T cells assessed by flow cytometry in HC (n=14, open circle) and SS (n=13, closed circle) patients. The results are shown as medians and interquartile ranges (IQRs). ***p≤0.001 compared with the HC group.

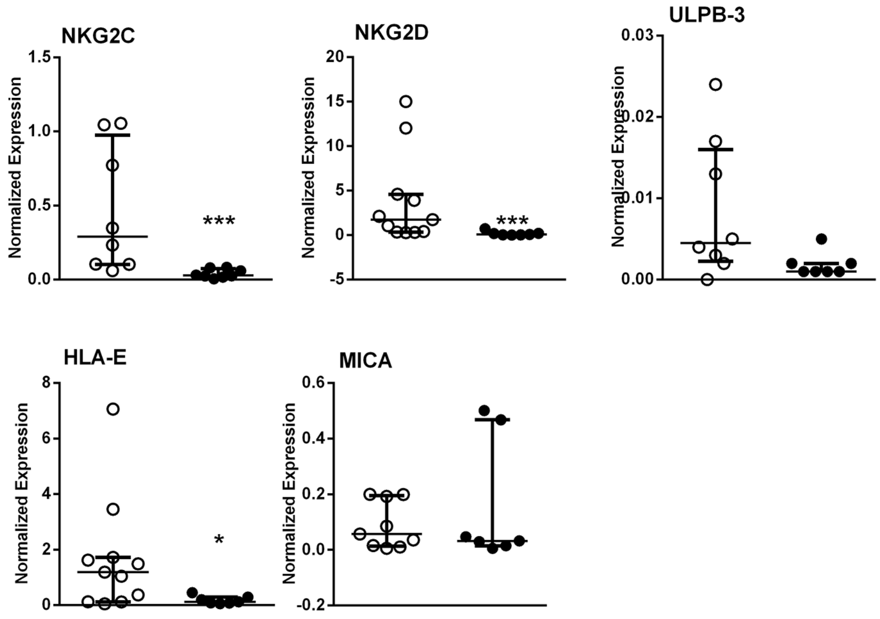

Supplementary Figure 3: Profile of the expression of NKG2C/D and their ligands in PBMCs from SS patients. A. Fresh PBMC from SS patients (n=7-8, closed circle) and HC subjects (n=9-11, open circle) was assessed for NKG2C/D, MICA, HLA-E and UBLP-3 mRNA expression by qPCR. The results are shown as medians and interquartile ranges (IQRs). $^{*}p \leq 0.05$, $^{***}p \leq 0.001$ compared with the HC group.

Supplementary Table 1: Laboratorial characteristics of Sézary syndrome patients

Patients	Age/Gender	CD4/mm^3	Sézary cells	CD4/CD8	CD4+ CD7- (%)	CD4+ CD26- (%)	LDH UI/L
**1**	75/F	9000	+	55.3	ND	75	1.19
**2**	55/F	20000	+	48	97	98	1.23
**3**	56/M	800	-	28	3	36	1.55
**4**	70/M	2000	+	31	ND	90	1.36
**7**	57/M	3100	+	18	82	74	1.74
**8**	68/M	6200	+	22	81	86	1
**9**	69/F	2300	-	6	23	81	1
**10**	56/M	6600	+	23	ND	79	1.84
**11**	62/M	8100	+	24	41	52	2.53
**15**	60/F	104000	+	24	99	99	1.7
**16**	65/F	3200	+	22.5	88	89	1.76
**19**	58/F	2000	+	10	2	61	1.57
**20**	69/F	4209	+	44	51	79	1.2

F= female, M= male; CD4+/mm^3 reference value 900-3400/mm^3; (+) = presence of Sézary cells≥1000/mm^3; ND= not done; LDH= lactate dehydrogenase.

Supplementary Table 2: Sequences of the primers used for qPCR

Gene	FW primer	REV primer
NKG2C	CCCCGAATACAAGAACGCAG	AGTACAGGCCAGCAAACTCT
NKG2D	CTGGTGAAGTCATATCATTGGATGG	GCTCGAGGCATAGAGTGCACAG
MICA	CAGACTGCCTGCAGGAACTA	TTTCTTCTTACAACAACGGACATA
HLA-E	TCTACCCTGCGGAGATCACA	TCGCTCCACTCAGCCTTAGA
ULPB-3	ATTCTTCCGTACCTGCTATT	GCTATCCTTCTCCCACTTCT

Supplementary Table 3: Genes analyzed for TLR-signaling pathway by PCR array

See Supplementary File 1
