# Supplementary material for: Profile of differentially expressed Toll-like receptor signaling genes in the natural killer cells of patients with Sézary syndrome
Source: Oncotarget. 2017 Sep 18;8(54):92183–94. doi: 10.18632/oncotarget.21006 (PMC5696173; doi:10.18632/oncotarget.21006)
Supplement: Supplementary file 2 [file oncotarget-08-92183-s002.docx]

| **Symbol** | **Description** | **Symbol** | **Description** |
| --- | --- | --- | --- |
| BTK | Bruton agammaglobulinemia tyrosine kinase | MAP2K4 | Mitogen-activated protein kinase 4 |
| CASP8 | Caspase 8, apoptosis-related cysteine peptidase | MAP3K1 | Mitogen-activated protein kinase 1 |
| CCL2 | Chemokine (C-C motif) ligand 2 | MAP3K7 | Mitogen-activated protein kinase 7 |
| CD14 | CD14 molecule | MAP4K4 | Mitogen-activated protein kinase 4 |
| CD180 | CD180 molecule | MAPK8 | Mitogen-activated protein kinase 8 |
| CD80 | CD80 molecule | MAPK8IP3 | Mitogen-activated protein kinase 8 interacting protein 3 |
| CD86 | CD86 molecule | MYD88 | Myeloid differentiation primary response gene (88) |
| CHUK | Conserved helix-loop-helix ubiquitous kinase | NFKB1 | Nuclear factor of kappa light polypeptide gene enhancer in B-cells 1 |
| CLEC4E | C-type lectin domain family 4, member E | NFKB2 | Nuclear factor of kappa light polypeptide gene enhancer in B-cells 2 (p49/p100) |
| CSF2 | Colony stimulating factor 2 (granulocyte-macrophage) | NFKBIA | Nuclear factor of kappa light polypeptide gene enhancer in B-cells inhibitor, alpha |
| CSF3 | Colony stimulating factor 3 (granulocyte) | NFKBIL1 | Nuclear factor of kappa light polypeptide gene enhancer in B-cells inhibitor-like 1 |
| CXCL10 | Chemokine (C-X-C motif) ligand 10 | NFRKB | Nuclear factor related to kappaB binding protein |
| ECSIT | ECSIT homolog (Drosophila) | NR2C2 | Nuclear receptor subfamily 2, group C, member 2 |
| EIF2AK2 | Eukaryotic translation initiation factor 2-alpha kinase 2 | PELI1 | Pellino homolog 1 (Drosophila) |
| ELK1 | ELK1, member of ETS oncogene family | PPARA | Peroxisome proliferator-activated receptor alpha |
| FADD | Fas (TNFRSF6)-associated via death domain | PRKRA | Protein kinase, interferon-inducible double stranded RNA dependent activator |
| FOS | FBJ murine osteosarcoma viral oncogene homolog | PTGS2 | Prostaglandin-endoperoxide synthase 2 (prostaglandin G/H synthase and cyclooxygenase) |
| HMGB1 | High mobility group box 1 | REL | V-rel reticuloendotheliosis viral oncogene homolog (avian) |
| HRAS | V-Ha-ras Harvey rat sarcoma viral oncogene homolog | RELA | V-rel reticuloendotheliosis viral oncogene homolog A (avian) |
| HSPA1A | Heat shock 70kDa protein 1A | RIPK2 | Receptor-interacting serine-threonine kinase 2 |
| HSPD1 | Heat shock 60kDa protein 1 (chaperonin) | SARM1 | Sterile alpha and TIR motif containing 1 |
| IFNA1 | Interferon, alpha 1 | SIGIRR | Single immunoglobulin and toll-interleukin 1 receptor (TIR) domain |
| IFNB1 | Interferon, beta 1, fibroblast | TAB1 | TGF-beta activated kinase 1/MAP3K7 binding protein 1 |
| IFNG | Interferon, gamma | TBK1 | TANK-binding kinase 1 |
| IKBKB | Inhibitor of kappa light polypeptide gene enhancer in B-cells, kinase beta | TICAM1 | Toll-like receptor adaptor molecule 1 |
| IL10 | Interleukin 10 | TICAM2 | Toll-like receptor adaptor molecule 2 |
| IL12A | Interleukin 12A (natural killer cell stimulatory factor 1, cytotoxic lymphocyte maturation factor 1, p35) | TIRAP | Toll-interleukin 1 receptor (TIR) domain containing adaptor protein |
| IL1A | Interleukin 1, alpha | TLR1 | Toll-like receptor 1 |
| IL1B | Interleukin 1, beta | TLR10 | Toll-like receptor 10 |
| IL2 | Interleukin 2 | TLR2 | Toll-like receptor 2 |
| IL6 | Interleukin 6 (interferon, beta 2) | TLR3 | Toll-like receptor 3 |
| CXCL8 | Interleukin 8 | TLR4 | Toll-like receptor 4 |
| IRAK1 | Interleukin-1 receptor-associated kinase 1 | TLR5 | Toll-like receptor 5 |
| IRAK2 | Interleukin-1 receptor-associated kinase 2 | TLR6 | Toll-like receptor 6 |
| IRAK4 | Interleukin-1 receptor-associated kinase 4 | TLR7 | Toll-like receptor 7 |
| IRF1 | Interferon regulatory factor 1 | TLR8 | Toll-like receptor 8 |
| IRF3 | Interferon regulatory factor 3 | TLR9 | Toll-like receptor 9 |
| JUN | Jun proto-oncogene | TNF | Tumor necrosis factor |
| LTA | Lymphotoxin alpha (TNF superfamily, member 1) | TNFRSF1A | Tumor necrosis factor receptor superfamily, member 1A |
| LY86 | Lymphocyte antigen 86 | TOLLIP | Toll interacting protein |
| LY96 | Lymphocyte antigen 96 | TRAF6 | TNF receptor-associated factor 6 |
| MAP2K3 | Mitogen-activated protein kinase 3 | UBE2N | Ubiquitin-conjugating enzyme E2N |

**Supplementary Table 3: Genes analyzed for TLR-signaling pathway by PCR array**

**RT^2^ ProfilePCR Arrays (QIAGEN)**
